# Alpha‐Gal Syndrome: An Underrated Serious Disease and a Potential Future Challenge

**DOI:** 10.1002/gch2.202300331

**Published:** 2024-06-03

**Authors:** Mengyuan Zhan, Jia Yin, Tengda Xu, Liping Wen

**Affiliations:** ^1^ Department of Allergy State Key Laboratory of Complex Severe and Rare Diseases Peking Union Medical College Hospital Chinese Academy of Medical Sciences and Peking Union Medical College Beijing 100730 China; ^2^ Allergy Department Beijing Key Laboratory of Precision Medicine for Diagnosis and Treatment of Allergic Diseases National Clinical Research Center for Dermatologic and Immunologic Diseases Peking Union Medical College Hospital Chinese Academy of Medical Sciences and Peking Union Medical College Beijing 100730 China; ^3^ Department of Health Care Peking Union Medical College Hospital Chinese Academy of Medical Sciences and Peking Union Medical College Beijing 100730 China

**Keywords:** alpha‐gal syndrome (AGS), cetuximab, delayed‐type allergy, mammalian meat allergy, red meat allergy, tick bites, ticks

## Abstract

Over the past decades, red meat allergy, also known as mammalian meat allergy, which manifests differently from classic food allergies, has been reported in different countries and regions, including China. The allergen of this disease is not a protein but an oligosaccharide: galactose‐α‐1,3‐galactose, i.e., alpha‐gal or α‐gal. Therefore, this clinical syndrome is also called α‐gal syndrome (AGS). It clinically manifests as delayed anaphylaxis, i.e., patients generally develop allergic symptoms 2–6 h after ingesting red meat. This clinical manifestation is believed to be related to sensitization to α‐gal after tick bites. Sensitized individuals may also develop anaphylaxis after ingesting food and medicine or being exposed to medical equipment containing α‐gal, such as cetuximab and gelatin. Here, the literature on AGS is reviewed for a better understanding of its pathogenesis, clinical diagnosis, and treatment.

## Graphical abstract

This review delineates alpha‐gal syndrome (AGS), a delayed hypersensitivity to red meat induced by the oligosaccharide alpha‐gal, commonly triggered by tick bites. Unlike conventional food allergies, AGS symptoms emerge several hours after ingestion and may be exacerbated by exposure to specific medical products. The article elaborates on the environmental triggers, underlying mechanisms, and contemporary strategies for diagnosing and managing the condition.

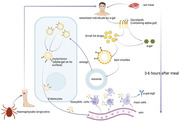

## 1 Introduction

Meat is an important protein source and can be roughly classified into red and white meat based on its uncooked visual characteristics. White meat, which encompasses poultry, seafood such as fish and shrimp, reptiles, etc., is characterized by its pale color when uncooked. Red meat, on the other hand, is derived from mammals, whose flesh appears red when uncooked due to the presence of myoglobin in the skeletal muscles. Red meat includes domestic meats, such as pork, beef, and lamb, and sometimes wild animals in certain regions, such as kangaroos, seals, and whales.^[^ ^1^ ^]^ The clinical presentations of mammalian meat allergy comprise two types of allergic reactions, immediate type and delayed type, although both are mediated by IgE antibodies. The immediate type manifests allergic symptoms shortly after the ingestion of meat and is primarily triggered by serum albumin and immunoglobulin, which are the major allergenic components. However, the delayed type is characterized by the onset of allergic symptoms 2–6 h after the ingestion of red meat and is mediated by oligosaccharide α‐gal‐specific IgE.^[^ ^1^, ^2^, ^3^, ^4^ ^]^ In tick‐endemic areas, the incidence of delayed allergy after consuming red meat was substantially higher than in nonendemic areas, and IgE antibodies against α‐gal were identified in the sera of patients with this delayed allergy.^[^ ^4^ ^]^

Alpha‐gal can be found in a variety of sources, including not only mammalian skeletal muscle but also mammalian visceral organs, fascia, smooth muscle, dairy products, and other products. Additionally, α‐gal can be present in gelatin, which is processed and produced from connective tissues such as mammalian bone and fascia, as well as in biological drugs and vaccines containing mammalian α‐gal.^[^ ^5^, ^6^, ^7^, ^8^ ^]^ In contrast to other allergic conditions, the allergen responsible for this particular syndrome, α‐gal, is not a protein but rather an oligosaccharide. In nature, this oligosaccharide is exclusively found in nonprimate mammals as well as certain primates, such as broad‐nosed monkeys, howler monkeys, spider monkeys, and other “New World monkeys”.^[^ ^4^ ^]^ Currently, the clinical manifestation of an allergic reaction to α‐gal is denoted as alpha‐gal syndrome (AGS) in the academic literature.

## 2 Galactose‐α−1,3‐Galactose (α‐gal)

The α‐gal[Supplementary-material gch21611-supitem-0001]epitope (Galα1‐3Galβ1‐4Glc NAC‐R) is abundantly expressed on glycoconjugates of protein‐linked glycan chains in nonprimate mammals, prosimians, and New World monkeys.^[^ ^5^, ^9^ ^]^ In contrast, α‐gal epitopes are not expressed in Old World monkeys, apes, or humans. The β‐galactose α−1,3‐galactosyltransferase (referred to as glycosyltransferase UDP‐Gal, α−1,3‐GT or α−1,3‐glycosidic bond transferase) was inactivated by deletion mutations during evolution, which prevented the formation of α−1,3‐glycosidic bonds. All immunocompetent humans can produce large quantities of natural anti‐α‐gal antibodies, including IgG and IgM, due to constant stimulation of bacterial antigens in the gut flora. These antibodies can precisely bind to α‐gal epitopes and elicit immunological rejection while exposed to α‐gal during medical interventions such as xenotransplantation.^[^ ^9^ ^]^

A major factor in human immunological rejection following mammalian xenotransplantation is natural α‐gal antibodies.^[^ ^10^ ^]^ The presence of mammalian α‐gal may cause failure of xenotransplantation by binding to anti‐α‐gal antibodies, mainly IgG antibodies, in the host and destroying the transplanted organ through complement and/or cellular pathways. It has been reported that xenotransplantation using α−1,3‐GT knockout pigs can substantially reduce immunological rejection because anti‐α‐gal antibodies act as an immune barrier to prevent transplantation of porcine organs into humans by binding to α‐gal epitopes expressed on porcine cells.^[^ ^11^ ^]^ Moreover, since anti‐α‐gal antibodies are naturally present in humans, they also provide novel approaches for the treatment of tumors; for example, endogenous experimental biological vaccines are targeted to α‐gal epitopes to enhance the immunogenicity of biological vaccines.^[^ ^12^ ^]^

Commins et al. determined that α‐gal‐specific IgE was the cause of delayed red meat allergy by investigating 24 patients who developed delayed urticaria or anaphylaxis after consuming beef, pork or lamb.^[^ ^2^ ^]^ Serum α‐gal‐specific IgE in these individuals responded to beef, pork, lamb, milk, and cat and dog dander but not chicken, turkey, or fish. Van Hage confirmed that the oligosaccharide previously identified as an IgE‐binding epitope on cat IgA was α‐gal.^[^ ^13^ ^]^ Since α‐gal epitopes are widely expressed in the cells and tissues of nonprimate mammals but not in human or monkey tissues, α‐gal‐specific IgE may be a primary allergen of mammalian meat; thus, alpha‐gal may be a primary allergen for allergy to a wide spectrum of mammalian meats in humans.^[^ ^4^ ^]^

## 3 Epidemiologic Studies have Revealed that Red Meat Allergy and AGS are Related to Tick Bites and Tick Distribution

An Australian allergist, Dr. Van Nunen, reported the first case series of delayed‐onset red meat allergy and found that most of these patients had a history of being bitten by ticks in the West Sydney jungle.^[^ ^3^ ^]^ Platts Mills determined that patients with red meat allergies had a fairly high incidence of α‐gal‐specific IgE positivity, and the geographic distribution of red meat allergy and elevated α‐gal‐specific IgE levels coincided with the prevalence of Rocky Mountain spotted fever (RMSF). Rocky Mountain spotted fever (RMSF) and *Amblyomma americanum* activity. This discovery suggested that further investigation into the potential function of tick bites in α‐gal sensitization was needed. A prospective study of three cases of tick‐bite reactions revealed that after tick bites, α‐gal‐specific IgE antibodies increased proportionally, which established a logical causal connection between them.^[^ ^4^ ^]^

At the 2011 Annual Meeting of the Chinese Allergy Association, we reported the first red meat allergy patient who developed delayed anaphylaxis after being bitten by ticks, identified as *Haemaphysalis longicornis*, and we published our first AGS study in 2015.^[^ ^1^, ^7^ ^]^ We measured sIgE to α‐gal antibodies and confirmed that the patient did have α‐gal sensitization, and western blotting confirmed the specific binding of animal meat and viscera (containing α‐gal) in the patient's serum.^[^ ^7^ ^]^ In recent years, we have published more AGS case series that include patients with IgE‐mediated hypersensitivity responses to alpha‐gal in red meat and vaccines.^[^ ^1^, ^6^, ^7^, ^8^ ^]^

The identification of α‐gal in tick gastrointestinal tracts in *Ixodes ricinus* was accomplished by Hamsten using an immunostaining method, which utilized mouse‐derived anti‐α‐gal polyclonal antibodies and sera of α‐gal‐specific IgE‐positive patients.^[^ ^14^ ^]^ Furthermore, Gary Crispell^[^ ^15^ ^]^ and Surendra Raj Sharma^[^ ^16^ ^]^ demonstrated the presence of α‐gal in the saliva of other ticks. In addition, Hamsten et al.^[^ ^17^ ^]^ reported a series of 39 patients with red meat allergy in Sweden; the majority of patients manifested anaphylaxis (37/39), and only two patients manifested mild clinical presentations (urticaria) after red meat ingestion. All patients were positive for α‐gal IgE and had a history of tick bites, and the majority of them reported tick bites more than 10 times.

However, the aforementioned studies could not explain why tick bites stimulate the human immune system to develop sIgE antibodies to this particular oligosaccharide. The currently prevailing theory is that tick bites play a critical role in red meat allergy: when ticks bite particular victims, they transfer α‐gal into the human immune system, which may change them from α‐gal IgE negative with red meat tolerance to α‐gal IgE positive with red meat allergy.^[^ ^18^ ^]^ However, the mechanism remains unknown. It has been hypothesized that the α‐gal in the tick digestive tract and salivary glands originates from nonprimate mammals that were previously bitten. After the α‐gal‐bearing tick attacks immune‐susceptible humans, α‐gal is injected into the victim and stimulates the victim's immune system to produce α‐gal‐specific IgE antibodies. It has been demonstrated that tick saliva contains α‐gal as well as bioactive molecules such as prostaglandin E2, which stimulates increased expression of anti‐inflammatory cytokines and decreases the production of proinflammatory mediators. Lots of studies have provided strong evidence indicating that tick bites are the cause of AGS in humans.^[^ ^2^, ^3^, ^4^, ^5^ ^]^ Yet in Maldonado‐Ruiz ‘s study,^[^ ^19^ ^]^ this theory has been challenged. They used an alpha‐galactosyltransferase knockout mutant mouse (aGT‐KO) model system infested with ticks that were unfed or partially fed on bovine blood. All of the treatments of aGT‐KO mice involving the feeding of partially fed and unfed ticks functioned as sensitizers that increased the levels of specific IgE against aGal. This study confirmed that aGT‐KO mice can be used as a model for RMA studies. However, these data, derived solely from animal experiments and not human trials, are not strong enough to contradict the tick “transmission” theory, as the results of this study show large individual variations. In our assessment, the critical factor is the degree of exposure to alpha‐gal. For instance, infrequent encounters such as a solitary tick sting (irrespective of the tick's prior activity) might be harmless. However, a rising frequency of tick bites potentially escalates the risk of AGS, considering that alpha‐gal can be synthesized by various species, including ticks. Given the complexity of this issue, further data are essential to reach a definitive conclusion. Taken together, these components may promote Th2‐associated immunity and stimulate α‐gal‐specific IgE production.^[^ ^20^, ^21^, ^22^ ^]^

Currently, ticks are extensively distributed across various regions of China, including species such as *Ixodes persulcatus*, *Haemaphysalis longicornis*, *Dermacentor silvarum*, *Hyalomma asiaticum*, *Rhipicephalus sanguineus*, and *Rhipicephalus microplus*.^[^ ^23^ ^]^ In regions where ticks are prevalent, it is advisable to provide extensive counseling to the patient and implement measures to safeguard individuals from both tick‐borne infectious diseases and AGS.

Compared to red meat allergy due to tick bites in Europe and the United States, studies of red meat allergy in China and Japan have shown that few of the patients with AGS noticed a history of tick bites, and some of them denied having been bitten by ticks or insects even after careful questioning.^[^ ^1^, ^6^, ^24^ ^]^ similar to what has been described in a South African cohort of patients described by Mabelane and Levin.^[^ ^25^ ^]^This may be related not only to a lack of effective scientific publicity and education but also to the relatively moderate or gentle tick bite pattern in Northeast Asia (identified as *Haemaphysalis longicornis* in both China and Japan), which differs from that of previous studies in Europe and the United States. This tick injects a variety of biologically active substances into the host simultaneously to facilitate its blood‐sucking, including proteins that anchor the mouthparts to the host's skin as well as enzymes; vasodilators; and anti‐inflammatory, hemostatic, and immunosuppressive substances; thus, the tick bite may be ignored due to absence of significant local pain or itching.^[^ ^24^ ^]^ This poses difficulties for both patient awareness and the clinical diagnosis.

Therefore, we recommend that if AGS is suspected, medical workers should pay more attention to α‐gal sensitization risk factors during collected medical history, including whether the patient lives in suburban, rural, pasture, or forested areas; whether they frequently go hiking in suburban or mountainous forested areas; whether there are large grassy shrubs or forests around their residence; whether they frequently move around these areas (especially during the spring and summer, when ticks are more active); and whether they have contact with outdoor pets, such as dogs or mammalian livestock such as pigs, horses, cattle, sheep, etc.^[^ ^1^ ^]^

## 4 Focus on Allergy or Anaphylaxis Due to Hidden α‐Gal Exposure

Cetuximab was the first medication discovered to be associated with AGS, although this novel clinical syndrome was not named AGS at that time.^[^ ^26^ ^]^ Cetuximab is an IgG1 subclass murine‐human chimeric monoclonal antibody used to treat epidermal growth factor receptor (EGFR)‐positive progressive or recurrent colorectal or head‐neck malignancies that cannot be surgically resected. A case report of cardiac arrest induced by an anaphylactic reaction associated with the first dose of cetuximab recently in 2022.^[^ ^27^ ^]^ Subsequently, there was also a case report of a patient who died of fatal anaphylaxis triggered by alpha‐gal syndrome after the first dose of cetuximab.^[^ ^28^ ^]^ According to these cases, AGS could cause fatal outcome undoubtedly. And these two cases were both reported in Japan, where *Haemaphysalis Iongicornis* is one of the dominant tick species just like in China. We suggest that sufficient attention should be given to all patients at risk of AGS and cetuximab allergy to avoid the catastrophic consequences. Investigators found a 22% incidence of allergic reactions in patients with a history of allergy following cetuximab injections in the southeastern United States, particularly in Tennessee and North Carolina, with many patients experiencing anaphylaxis during or soon after their first cetuximab injection.^[^ ^29^ ^]^ In addition, 20.8% of healthy control subjects in Tennessee were positive for IgE antibodies to cetuximab, according to Chung et al.^[^ ^26^ ^]^ Subsequent research indicated that the mechanism by which cetuximab causes anaphylaxis may be related to the presence of specific IgE antibodies in patients against the oligosaccharide α‐gal epitope on the Fab fragment in cetuximab.^[^ ^26^ ^]^ Allergic reactions to cetuximab usually manifest during or within 20 min of infusion, are severe and progress rapidly, and can even result in death.^[^ ^27^, ^30^ ^]^ Once the patients were sensitized, the clinical manifestations were so severe that they had to discontinue further usage of cetuximab for life.^[^ ^26^, ^27^, ^28^ ^]^

The etiology of cetuximab‐induced anaphylaxis is the presence of α‐gal‐specific IgE antibodies in patients. Cetuximab‐induced anaphylaxis in patients with AGS is due to the presence of oligosaccharide alpha‐gal, found at the 88th amino acid of the Fab portion of the cetuximab heavy chain.^[^ ^26^ ^]^ Cetuximab anaphylaxis has been shown to affect only patients with α‐gal‐specific IgE antibodies to the Fab segment of cetuximab.^[^ ^26^, ^31^ ^]^

Gelatin is a complex combination of proteins and peptides produced through partial hydrolysis of collagen extracted from the skin, bone, or connective tissue of bovine or porcine animals or fish.^[^ ^8^ ^]^ Gelatin is widely utilized as a food ingredient or additive,^[^ ^32^ ^]^ a substitute for colloidal plasma in medical usage,^[^ ^33^, ^34^ ^]^ and a stabilizer in vaccines.^[^ ^35^ ^]^ The majority of patients allergic to red meat were also sensitized to gelatin, with a subset showing clinical allergies to both. The presence of α‐Gal in gelatin and a correlation between α‐Gal and gelatin test results suggest that α‐Gal IgE may be the reactive target to gelatin. However, the pathogenic link between tick bites and sensitization to red meat, α‐Gal, and gelatin (with or without clinical reactivity) remains unclear.^[^ ^36^ ^]^

Numerous cases of allergy or anaphylaxis to gelatin‐containing food,^[^ ^32^ ^]^ vaccines,^[^ ^37^, ^38^ ^]^ and intravascular volume expanders,^[^ ^33^, ^34^, ^39^ ^]^ and gelatin‐based hemostatic agents have been reported.^[^ ^40^, ^41^ ^]^ The duration of onset depends on the route by which gelatin was introduced. Our 2018 study revealed significant age differences in patients with allergies to different allergens: gelatin vaccine allergies are mainly seen in young children, while gelatin injection allergies occur more frequently in middle‐aged and elderly adults. This pattern reflects the different medical interventions typical for these age groups, with vaccines primarily given to children and gelatin‐based fluids more commonly used in surgeries for adults.^[^ ^8^ ^]^

The fastest onset is caused by exposure to a hemostatic sponge containing gelatin when filling a wound during intraoperative hemostasis, which usually manifests 5 min after exposure, followed by intravenous infusion of gelatin as a colloidal capacity supplement. For suppositories containing gelatin, allergic reactions may occur 0.5 h afterward, slightly longer than after intravenous preparations. Allergy symptoms caused by gelatin in vaccines usually occur 0.5–1 h after vaccine injection.^[^ ^8^ ^]^ Gelatin in vaccines for measles mumps and rubella (MMR), chickenpox, encephalitis, rubella, and influenza virus has been documented to cause allergies, with MMR being the most prevalent.^[^ ^8^, ^37^ ^]^

The slowest onset is through oral exposure. Ingestion of food such as gummy or candy containing gelatin can trigger allergic symptoms after several hours. The reason is that more time is required for digestion and decomposition in the gastrointestinal tract, and digestion may lead to a decrease in the content of gelatin components and a decrease in allergenicity. We previously reported anaphylaxis caused by gelatin components in chickenpox and hepatitis A vaccines.^[^ ^8^ ^]^ Young children are prone to allergies to gelatin in vaccines, while middle‐aged or elderly adults are prone to gelatin infusion allergies. The reason for this difference is related to the different medical interventions received by different age groups.^[^ ^8^ ^]^ Vaccination is a preventive measure for diseases and is mainly used in young children. Gelatin rehydration is mainly used for the prevention and treatment of hypovolemia and is mostly used in the perioperative period.

Existing evidence suggests that IgE to alpha‐gal can be induced by tick bites or associated with tick bites. If further tick bites are carefully avoided, allergic reactions in some patients with red meat allergies may subside over time.^[^ ^42^ ^]^ It is important to note, however, that the absence of allergic reaction to consumed red meat is not a guarantee that the patient will not react to α‐gal‐containing medications during the perioperative period.^[^ ^43^ ^]^ Evaluation of sIgE antibodies to α‐gal is recommended in high‐risk patients prior to surgery to reduce the risk of anaphylaxis.

## 5 Blood Type is Connected to the Prevalence of AGS

Nearly all nonprimate mammals express the epitope of glycan galactose‐α−1,3‐galactose (α‐gal) on protein‐linked glycan chains, which are structurally similar to human blood group B substances.^[^ ^4^, ^44^ ^]^ In xenotransplantation, this glycan is a well‐recognized immune barrier.^[^ ^10^, ^44^ ^]^

Human blood type was associated with the prevalence of red meat allergy under an equivalent exposure density of tick bites. Hamsten et al. analyzed 39 Swedish patients with a history of red meat allergy, 37 of whom had non‐B blood types (A or O).^[^ ^17^, ^45^ ^]^ Similar results were discovered in a Chinese clinical investigation.^[^ ^6^ ^]^ It is suggested that individuals of types A or O are more likely to produce anti‐α‐gal‐specific IgE antibodies following tick bites, whereas individuals of types B and AB are more likely to develop tolerance to α‐gal antigens and are therefore less susceptible to red meat allergy following tick bites. The B antigen protects against the development of red meat allergy.^[^ ^45^ ^]^ The exact mechanism is unknown, but multiple current speculations have been reported.^[^ ^6^, ^45^ ^]^

**Figure 1:**
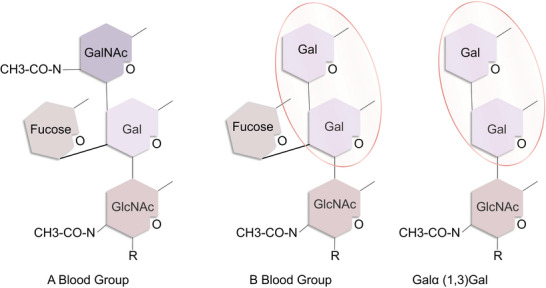
ABO blood group and alpha‐gal simple structure diagram. The B antigen structure is similar to the core structure of the α‐gal epitope, with a nonreducing end of disaccharide galactose‐1,3‐galactose (gal‐1,3‐gal), so individuals with the B and AB blood groups who have the B antigen structure are more likely to tolerate alpha‐gal [this similar part of the structure has been circled in the oval box]. (Figure 1 is created by Mengyuan Zhan, this figure references Figure 1 from the article ‘ABO Blood Groups and Related Antigens, Natural Antibodies, and Transplantation’ by M. S. Sandrin et al.).

Human ABO blood group substances, primarily oligosaccharides, are expressed on the membrane of erythrocytes. Human type B antigens are produced by combining an α‐gal structure with a fucose residue. Individuals of blood type B have an oligosaccharide structure comparable to that of α‐gal in nonprimate mammals; therefore, their immune systems are more likely to perceive α‐gal as self and are more resistant to sensitization to α‐gal (**Figure** **1**). Thus, it is suggested that blood B antigens may play a protective role against red meat allergy.^[^ ^44^ ^]^

To date, conclusive scientific evidence regarding the link between ABO blood type and red meat allergy or AGS syndrome has not been published. Different blood types may carry different susceptibilities to a certain food allergy. Alejandro Cabezas‐Cruz's research indicates that blood‐type antigens influence the immune system's capacity to generate anti‐α‐gal Abs (IgM and IgE), thereby influencing an individual's susceptibility to AGS.^[^ ^46^ ^]^ Current observations suggest that blood type B may reduce the immune system's capacity to produce anti‐α‐gal antibodies, presumably due to its tolerance to α‐gal, the structure of which closely resembles that of antigen B. Therefore, individuals with reduced levels of blood type B and anti‐α‐gal Abs are less likely to develop AGS.

## 6 Clinical Advances in the Diagnosis and Treatment of AGS

Red meat allergy is characterized by recurrent acute urticaria or anaphylaxis. Clinical manifestations include: cutaneous (itching, urticaria with or without angioedema), gastrointestinal (nausea, vomiting, abdominal pain, diarrhea), respiratory (dyspnea), cardiovascular (palpitations, hypotension), and neurologic (dizziness, anxiety, feeling of impending death, loss of consciousness). Clinical manifestations^[^ ^2^, ^6^ ^]^ Patients typically visit the Emergency Room (ER) at midnight, e.g., 11 pm to 2 am, due to the unique characteristics of delayed anaphylaxis, which is induced by consuming red meat at supper several hours before the attack.^[^ ^1^, ^6^ ^]^ The clinical manifestations of different patients varied, and even the manifestations in the same patient varied depending on the quantity and quality of the alpha‐gal product ingested, fatty cuts of meat are frequently associated with delayed AGS symptoms, while some patients may tolerate small amounts of lean cuts of meat.^[^ ^42^ ^]^ Not all exposure to red meat results in severe clinical reactions, yet moderate or severe allergic symptoms occur in the majority of cases. It is indeed crucial for individuals sensitive to alpha‐gal to be mindful not only of avoiding mammalian meat, including offal, but also of alpha‐gal‐containing drugs (e.g., cetuximab), gelatin used in intravascular volume expanders, and sometimes even dairy products and gelatin‐containing foods such as gummy candies.^[^ ^1^, ^6^, ^7^, ^8^ ^]^

Not all patients who have experienced tick bites will develop α‐gal sensitization, i.e., AGS or red meat allergy. At present, the risk factors for AGS are as follows:

1) Frequency and density of tick exposure: Current research has indicated that the prevalence of α‐gal sensitization increases proportionally with the number of tick bites.^[^ ^47^ ^]^
2) Interval between ticks biting nonprimate mammals and biting humans and the amount of residual α‐gal in ticks' gastrointestinal or salivary glands.^[^ ^48^ ^]^
3) Blood type of individuals who suffer from tick bites: The immune system of individuals with B and AB blood types is prone to recognize α‐gal as autogenic and thus less likely to induce AGS.^[^ ^46^ ^]^
4) Atopy: Kiewiet et al. suggested that more than half of AGS patients were atopic, which may increase the risk of anaphylaxis, especially respiratory manifestations.^[^ ^49^ ^]^

Yet there are equally studies that demonstrate that you do NOT have to have other signs of atopy in order to be sensitized to alpha‐gal.^[^ ^50^ ^]^

5) Fat content in mammalian meat consumed by humans: A higher fat content can lead to more severe allergic manifestations, which indicates a synergistic effect between fat content and the prevalence of AGS.^[^ ^42^ ^]^
6) Individuals with systemic mastocytosis or a history of idiopathic anaphylaxis may be at a higher risk for alpha‐gal‐induced anaphylaxis.^[^ ^51^ ^]^

**Figure 2:**
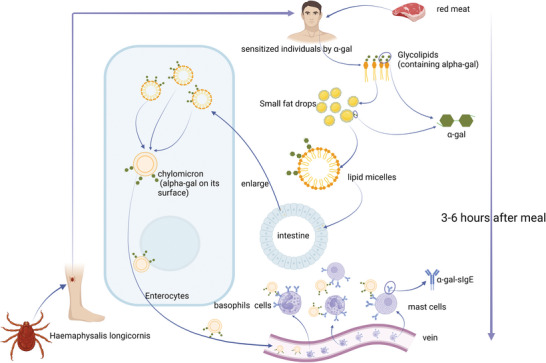
Speculation of the lipid digestion mechanism. Red meat (from pigs, cows, and sheep) is the main source of alpha‐gal in the human diet. Red meat digestion forms lipid micelles from glycolipids containing alpha‐gal in tick‐sensitized people. Small intestinal digesting enzymes (primarily pancreatic lipase) hydrolyze micelle triglycerides into free fatty acids, mono‐ and diglycerides, which intestinal cells absorb. The intestinal epithelium forms chylomicron, which expose α‐gal molecules. After entering the lymph through the lacteal vein, the α‐gal molecules cross‐link with basophil or mast cell surface α‐gal‐specific IgE antibodies in the subclavian venous blood ≈4 h after eating, causing allergic reactions or anaphylaxis. Reproduced with permission^[^ ^20^ ^]^ this figure is distributed under the Creative Commons Attribution License (CC BY).

Red meat allergy usually manifests in a delayed pattern, different from traditional IgE‐mediated food allergy, i.e., allergic symptoms typically begin 2 to 6 hours after meat consumption. However, some patients reported a shorter delay, which was typically associated with alcohol consumption, high α‐gal content in food, and exercise after a meal.^[^ ^52^ ^]^ It is currently believed that the delayed symptoms of red meat allergy are related to the digestion, absorption, and transfer of related glycoproteins and/or glycolipids. Compared to proteins, lipids are digested through a distinct, slower mechanism. A recent in vitro study indicated that only α‐gal that bonds to lipids can travel through a single layer of intestinal epithelial cells and activate basophil granulocytes in individuals with α‐gal allergies.^[^ ^53^ ^]^ Since lipids are digested and absorbed more slowly than proteins, the combination of α‐gal and lipids may induce delayed reactions in patients with α‐gal allergies (**Figure** **2**).

The diagnosis of red meat allergy is based on the delayed onset of allergic reaction or anaphylaxis after intake of red meat, proven sensitization to red meat (skin test and/or serum IgE), and α‐gal‐sIgE ≥ 0.10 Ku L^−1^ (Detection was performed using ImmunoCAP by Thermo Fisher Scientific, Uppsala, Sweden).^[^ ^42^ ^]^ The absence of tick bites does not exclude the diagnosis since tick bites might be neither painful nor itching and thus ignored by the patients. Currently, diagnosis of red meat allergy is difficult and challenging, partly due to the delay of symptoms. Percutaneous skin prick testing of red meat in alpha‐gal syndrome can often lead to false negatives. Prick testing using alpha‐gal‐containing reagents such as cetuximab and gelatin, or fresh prick to prick testing with raw mammal meat can reduce the risk of false negative results. The sensitivity of intradermal tests of red meat is higher than that of prick tests yet no longer available in most countries since it may induce anaphylaxis. Currently, the US Centers for Disease Control (CDC) states that the appropriate diagnostic tests for trying to confirm a diagnosis of alpha‐gal syndrome is the alpha‐gal specific IgE ImmunoCAP test and NOT percutaneous skin prick testing. A prick test is typically only considered if the alpha‐gal IgE in serum is negative but the clinical history is highly suspicious for alpha‐gal syndrome.^[^ ^54^, ^55^ ^]^

Detection of α‐gal sIgE is crucial to the diagnosis of AGS. We detected α‐gal sIgE in patients with clinically confirmed red meat allergies, all of whom were α‐gal sIgE positive.^[^ ^1^, ^6^, ^7^, ^8^ ^]^ When α‐gal sIgE is negative, a test for feline serum albumin‐specific IgE (Feld 2) may be useful in identifying patients with suspected pork cat syndrome.^[^ ^56^ ^]^ Basophil activation tests based on CD63 expression can also be considered.^[^ ^57^, ^58^, ^59^ ^]^ Sensitization to dogs or cats may serve as collateral evidence of red meat allergy or AGS since all of our patients tested positive for dog and/or cat dander. Based on our experience, the semiquantitative allergen panels currently available in China exhibit rather poor accuracy in detecting red meat allergy [unpublished data].

Studies have shown that exposure to α‐gal transmitted by tick bite may cause the affected individual to produce both α‐gal‐specific IgG and IgE. The likelihood of developing a transition from α‐gal IgG to IgE increases with the frequency of tick bites.^[^ ^60^ ^]^ It is unclear how and when this conversion occurs. Aejandro Joral et al. measured α‐gal sIgG and IgE levels using the ImmunoCap Assay System to determine the risk of AGS in subjects who had been bitten by ticks but did not have AGS yet. He suggested that the α‐gal IgG level could play a role as a predictive factor for developing AGS. The research showed that when α‐gal‐specific sIgG in individuals bitten by ticks was ≥40 µg mL^−1^, the risk of developing AGS was ≈35%.^[^ ^60^ ^]^

In individuals with a history of tick bites but no AGS, IgE levels may correlate with the risk of developing AGS in the future. Mabelane et al.^[^ ^25^ ^]^ noted that the level of α‐gal sIgE and the ratio of α‐gal sIgE to total IgE correlated with the possibility of developing AGS and red meat allergy. There was a 95% probability of meat allergy if α‐gal sIgE was higher than 5.5 kU L^−1^ and the ratio was higher than 2.12%. A recent study showed that an α‐gal sIgE titer ≥ 0.54 KUa mL^−1^ was predictive of AGS.^[^ ^62^ ^][^ ^61^ ^]^ However, the positive predicted values in both studies may need to be validated by more research and data.^[^ ^62^ ^]^ The level of α‐gal sIgE is elevated after a tick bite and may decrease after strict tick bite avoidance.^[^ ^63^ ^]^ Thus, prevention of further tick bites may potentially prevent the development of red meat allergy even in individuals with a past history of tick bites. However, there are significant individual differences in the correlation between clinical manifestations of AGS and α‐gal sIgE titers. In some patients, symptoms improve after the avoidance of tick bites, and red meat can be gradually tolerated. However, many patients are unable to consume red meat for life, regardless of whether they avoid tick bites, the mechanism of which remains unclear. It is currently believed that people with AGS could continue to have pets such as dogs and cats, as there is no causal relationship between keeping their pets and persistent allergy to red meat.^[^ ^52^ ^]^

Although the titer of α‐gal sIgE or the ratio of sIgE to total IgE may correlate with the possibility of developing AGS after tick bites, it cannot predict the severity of the symptoms.^[^ ^42^ ^]^ The amount of red meat or α‐gal intake and other cofactors, such as concomitant alcohol consumption and exercise after meals, can also affect the timing of attacks and the severity of clinical symptoms. According to the clinical experience of food‐dependent exercise‐induced anaphylaxis (FDEIA) or wheat‐dependent exercise‐induced anaphylaxis (WDEIA), the presence of cofactors appears to reduce the dietary dose required to induce symptoms, and this phenomenon should be explored further in AGS.^[^ ^64^ ^]^ Recent tick bites appear to increase patients' sensitivity to previously tolerated red meat exposures, which is indicated by a lower threshold of the food challenge test.^[^ ^18^, ^42^ ^]^

With further understanding of α‐gal, red meat (beef/pork/lamb) allergy, or mammalian meat allergy is gradually being more accurately described as α‐gal allergy.^[^ ^6^ ^]^ In addition to cetuximab and red meat, it is recognized that these patients would be at risk of allergic reaction or anaphylaxis after exposure to other medication or foods containing α‐gal epitopes.^[^ ^32^, ^65^ ^]^ Examples include the extraction of pharmaceutical‐grade porcine (intestinal) or bovine (lung) heparin,^[^ ^66^ ^]^ bioartificial cardiac valves,^[^ ^10^, ^65^ ^]^ gelatin, or stearic acid.^[^ ^8^, ^67^ ^]^ It is suggested that patients with confirmed red meat allergy undergo a heparin skin prick test and intradermal test before receiving medication or undergoing surgical procedures. The intradermal test has a higher sensitivity than the skin prick test. According to our experience, the prick test should be performed first, and if the results are negative, the intradermal test should be performed, along with a positive control (histamine) and a negative control (saline). In addition, it is recommended to use the same batch of heparin in the skin test that will be used in the surgery.^[^ ^66^, ^68^ ^]^

Recently, Péter Apari et al.^[^ ^69^ ^]^ examined the relationship between red meat allergy and tumor immunity and proposed that AGS is an adaptive cancer defense mechanism. Paleontological studies indicate that the α‐gal gene and Neu5Gc (N‐hydroxyneuraminic acid) gene were lost from the human genome 30 and 2 million years ago, respectively, during evolution. Loss of α‐gal, which may have enhanced human immunity against infections (e.g., viruses, bacteria, and protozoa), and loss of Neu5Gc, which occurred almost simultaneously with humans learning to use fire to cook meat, may have reduced the risk of various cancers (e.g., stomach, colorectal) associated with the transition from raw to cooked meat in the human diet. Through tick infections and ingestion of mammalian meat, humans acquire α‐gal, and Neu5Gc from external sources, which accumulate in latent tumor tissue, leading to tumorigenesis and development. This hypothesis, if tested further, could be significant in the search for novel molecular tumor markers and for candidate molecules of immune attack effects on tumor cells (e.g., Neu5Gc^[^ ^70^ ^]^) and may provide a new approach for cancer research and treatment.

## 7 Summary

AGS is an unusual clinical syndrome characterized by α‐gal sensitization, which means the presence of α‐gal specific IgE, and may manifest as delayed food allergy or anaphylaxis after consuming mammalian meat. If exposed to medications, vaccines, or medical instruments containing α‐gal through oral, injection, transplantation, or surgical routes, sensitized individuals are at risk of developing urticaria, anaphylaxis, or even death. Tick bites are regarded as an important sensitization pathway, particularly in populations with blood groups A or O (specifically, non‐B type). Currently, the pathogenesis of AGS is not completely understood. Further research on the pathogenesis of AGS will provide a deeper understanding of the pathogenesis, diagnosis, and treatment of infection, allergic diseases, and even tumors.

## Conflict of Interest

The authors declare no conflict of interest.

## Supporting information

Supporting Information
