# Supplementary material for: Alpha‐Gal Syndrome: An Underrated Serious Disease and a Potential Future Challenge
Source: Glob Chall. 2024 Jun 3;8(7):2300331. doi: 10.1002/gch2.202300331 (PMC11237182; doi:10.1002/gch2.202300331)
Supplement: Supplementary file 1 — Supporting Information [file GCH2-8-2300331-s001.docx]

**Supplementary materials：**

The supplementary materials predominantly comprise the original Chinese literature referenced in this article, accompanied by their English abstracts. Within these, three Chinese references, specifically references 6, 7, and 8, pertain to the study of red meat and gelatin allergies in China. Furthermore, Our first case report on red meat allergy in China was presented in 2011 at an annual meeting, details of which can be found in the supplementary materials(reference0). This case was subsequently published in an English-language journal, Allergy, Asthma, and Immunology Research (AAIR), in 2015, as referenced in Reference1.

--------------------------------------------------------------------------------------------------------------------------------------------------------------------------------------------------------------------------------------------

**The English abstracts of these four references in Chinese are given below for your reference:**

Reference6

[6] L. Lisha, W. Liping, Y. Jia, Z. Junxiong, G. Jianqing, L. Hong, W. Lianglu, G. Kai, S. Jinglv,Q. Rui *Chin J Allergy Clin Immunol.* **2018,** *12,* 522-526.

**Title: Clinical characteristics of mammalian meat allergy**

Abstract：

Objective ：Mammalian meat allergy is one kind of delayed-onset IgE-mediated allergic reaction after ingestion of mammalian meat，and its related allergen epitope is a carbohydrate moiety galactose-α-1，3-galactose ( α-Gal ) ． The clinical history and results of assistant examinations of 13 Chinese cases of mammalian meat allergy were analyzed and clinical features were also summarized in this study.

Methods ：Suspected cases of mammalian meat allergy were collected from Department of Allergy，Peking Union Medical College Hospital． The clinical history was recorded; skin prick tests，intradermal tests and serum specific immunoglobulin E ( sIgE) tests were performed．

Results ：Diagnosis of mammalian meat allergy was confirmed among 13 patients，whose median course of disease was 35 months． Delayed onset of allergic symptoms was reported by most patients，which were defined as manifestations occurring 3-5 hours after the ingestion of mammalian meat． Only one patient described the rapid onset of symptoms，only 10 minutes after eating meat． The most common symptoms were urticaria and cutaneous itching ( 100%) ． Nine patients ( 69. 2%) were attacked by dyspnea or wheezing，and also nine patients ( 69. 2%) were suffering from vomiting，diarrhea or abdominal pain，while eight patients ( 61. 5%) with palpitation or syncope. 84. 6% of patients belonged to the B-negative blood groups ( type A and type O) ，and only 38. 5% with history of tick bites． Positive results of mammalian meat skin prick tests were shown among 66. 7% of cases，while positive results of intradermal tests were shown among 100% patients． Positive pork-and α-Gal-sIgE detection results were shown among 84. 6% and 76. 9% of patients respectively．

Conclusions ：The clinical features of 13 Chinese patients with mammalian meat allergy were

summarized first time in China，whereas partial clinical features were different from published literatures． Most patients denied history of tick bites and one cases reported typical immediate allergic reaction，whose onset of symptoms was within 10 minutes after eating the mammalian meat in our group． Further investigation should be done to clarify the potential existence of another epitope besides α-Gal which was related to immediate allergic reaction after ingestion of mammalian meat．

--------------------------------------------------------------------------------------------------------------------------------------------------------------------------------------------------------------------------------------------

Reference7

[7] W. Liping, Z. Junxiong, Y. Jia, L. Hong, S. Jinlv, W. Ruiqi,S. Yi. Chin J Allergy Clin Immunol. 2015, 9, 1-7+81-82.

**Title: Clinical Case Study on Anaphylaxis Induced by Red Meat Ingestion**

Abstract：

Objective：A novel delayed anaphylactic reaction to red meat，associated with tick bites and IgE

antibodies against galactose-α-1，3-galactose ( α-gal) ，was reported in Australia，US，and Europe since 2009. This is the first clinical case study of red meat induced delayed anaphylaxis in China. Method：Two cases of delayed anaphylactic reactions to red meat were reported for the first time in China. Clinical manifestations were summarized，allergens were investigated，and questionnaire about tick bite was taken as well. Analysis of the antigen-binding characteristics of serum specific IgE antibodies with pork，innards were performed，based on western blotting by using sera from case 1 and a healthy control to observe the features of specific binding characteristics.

Results：The two cases ( case 1 female57 yrs，case 2 male44 yrs) are both farmers who live in the suburb of Beijing，manifested with anaphylactic reactions three to six hours after red meat ingestion. Serum specific IgE and skin test results to multiple mammalian proteins were positive. Specific IgE to galactose-α-1，3-galactose were positive in the two patients ( ＞ 100 kUL in case 1 and 6. 00 kUL in case 2)．Case 1 reported a history of tick biting with large regional reactions 2-3 weeks before anaphylaxis. The ticks on her dog were captured and authenticated as hematophagous ixodidae. Case 2 did not report history of tick bites. The increased concentration of IgE reactive epitopes in mammalian organs was confirmed by using western blotting with serum of case 1.

Conclusions：Ingestion of red meat can induce severe and sometimes life-threatening clinical manifestations，which might be induced by tick bite. This issue should arouse concerns both in public and doctors. Patients with red meat allergy should avoid intake of meat as well as innards of non-primate mammalian.

--------------------------------------------------------------------------------------------------------------------------------------------------------------------------------------------------------------------------------------------

Reference8

[8] L. Lisha, W. Liping,G. Kai *Chin J Allergy Clin Immunol.* **2018,** *12,* 409-416.

**Title: Gelatin-contained vaccine allergy induced anaphylaxis: two cases report and literature**

**review**

**Abstract**

Objective：Two Chinese cases of gelatin-contained vaccine induced anaphylaxis were reported and the clinical features of immediate allergic reaction to gelatin were summarized basing on literatures.

Methods：The characteristics of patients with immediate allergic reaction to gelatin was reviewed in this article based on the search results of multiple domestic and international databases.

Results：Two cases of pediatric patients with immediate systemic allergic reaction to gelatin-contained vaccines were first reported in China． Fifty-nine articles in English and another 36 in Chinese were eligible in the past 50 years and reviewed in this paper，including 380 patients with immediate hypersensitivity to gelatin. 55. 0% of them were induced by gelatin-contained vaccines and 30. 5% by gelatin-contained plasma substitute. 86. 2% of gelatin-contained plasma substitute-induced systemic allergic reaction was graded as severe reaction，which was significantly higher than that induced by gelatin-contained vaccines ( 27.3%) or gelatin-contained food ( 33.3%). The positive rate of gelatin skin test and serum IgE detection results were 96.3% and 48.1%，respectively，among the patients who were prescribed both in vivo and in vitro tests.

Conclusions：Severe systemic allergic reaction is more likely induced by gelatin-contained plasma substitutes in compared to gelatin-contained vaccines or food. The sensitivity of skin test is higher than that in vitro sIgE detection as assistant diagnostic methods for gelatin allergy.

--------------------------------------------------------------------------------------------------------------------------------------------------------------------------------------------------------------------------------------------

Reference0

**Title: An Instance of Severe Allergic Reaction Caused by Pork Allergy**

Abstract：

**Objective**: To precisely identify the allergen in a patient with a severe allergic reaction and to initially explore the diagnostic methods for delayed anaphylactic shock caused by this rare food allergy.

**Methods**: Detailed history collection and serial follow-ups were conducted, along with allergen skin tests and serum laboratory examinations.

**Results**: The skin tests showed strong positive reactions to pork, beef, and lamb. The patient had a positive reaction to pork and beef. Through detailed history collection and follow-ups, it was observed that accidental consumption of a small amount of pork during the follow-up period triggered a systemic urticaria, respiratory distress, and hypotension. Strict dietary control subsequently prevented similar episodes.

**Conclusion**: Serum and skin testing are useful in diagnosing this rare disease. This case report is the first of its kind to be reported in the country.
